# A Fair Channel Hopping Scheme for LoRa Networks with Multiple Single-Channel Gateways

**DOI:** 10.3390/s22145260

**Published:** 2022-07-14

**Authors:** Alexandre Figueiredo, Miguel Luís, André Zúquete

**Affiliations:** 1 Instituto de Telecomunicações, 3810-193 Aveiro, Portugal; alex.figueiredo@ua.pt (A.F.); andre.zuquete@ua.pt (A.Z.); 2 ISEL—Instituto Superior de Engenharia Lisboa, Instituto Politécnico de Lisboa, 1959-001 Lisbon, Portugal; 3 Departamento de Eletrónica, Telecomunicações e Informática (DETI), University of Aveiro, 3810-193 Aveiro, Portugal

**Keywords:** low power wide-area networks, large-scale LoRa networks, single-channel LoRa gateways

## Abstract

LoRa is one of the most prominent LPWAN technologies due to its suitable characteristics for supporting large-scale IoT networks, as it offers long-range communications at low power consumption. The latter is granted mainly because end-nodes transmit directly to the gateways and no energy is spent in multi-hop transmissions. LoRaWAN gateways can successfully receive simultaneous transmissions on multiple channels. However, such gateways can be costly when compared to simpler single-channel LoRa transceivers, and at the same time they are configured to operate with pure-ALOHA, the well-known and fragile channel access scheme used in LoRaWAN. This work presents a fair, control-based channel hopping-based medium access scheme for LoRa networks with multiple single-channel gateways. Compared with the pure-ALOHA used in LoRaWAN, the protocol proposed here achieves higher goodput and fairness levels because each device can choose its most appropriate channel to transmit at a higher rate and spending less energy. Several simulation results considering different network densities and different numbers of single-channel LoRa gateways show that our proposal is able to achieve a packet delivery ratio (PDR) of around 18% for a network size of 2000 end-nodes and one gateway, and a PDR of almost 50% when four LoRa gateways are considered, compared to 2% and 6%, respectively, achieved by the pure-ALOHA approach.

## 1. Introduction

The emergence of the Internet of Things (IoT) [1,2], potentiated by its ability to connect every device to the Internet, increased the demand for low power wide-area networks (LPWANs). LPWANs are a category of wireless communication technologies with unique characteristics, such as long-range communications, low power consumption, and low deployment costs, making them suitable for IoT applications. Therefore, to satisfy the wide range of IoT applications, several communication technologies were developed, such as LoRa, Sigfox [3], Ingenu [4], and NB-IoT [5].

Long-range (LoRa) [6] has been one of the most popular and exciting LPWAN technologies thanks to its robustness to noise, which allows long-range transmissions, the non-destructive property of colliding packets, and its power efficiency. LoRa, initially only a physical layer, was extended by adding a medium access control (MAC) layer, LoRaWAN, standardized and open-sourced by the LoRa Alliance. This MAC layer defines the network architecture, along with a channel access scheme and the adaptive data rate (ADR) mechanism. This mechanism aims to optimize the transmissions by dynamically changing the transmission parameters according to the signal quality with the receivers.

LoRa operates on the sub-GHz Industrial, Scientific and Medical (ISM) unlicensed bands, the 433 and 868 MHz bands in Europe. These bands are free to use and have less retention than other bands, such as the 2.4 and 5 GHz bands. However, in most situations, the transmissions are limited to 1% of the duty cycle. LoRa signals are modulated using chirp spread spectrum (CSS) modulation, which provides excellent resistance to noise. LoRa allows the customization of several transmission parameters, such as spreading factor (SF), coding rate (CR), bandwidth (BW), and transmission power (TP), resulting in different and orthogonal LoRa communication channels, with specific communication ranges, robustness, and data rates. For example, a larger SF improves the communication range but also increases the time on air (ToA), which increases the energy consumption and reduces the data rate.

Multiple-channel LoRaWAN GWs can simultaneously receive multiple packets with different spreading factors; however, they can be expensive and operate according to the pure-ALOHA channel access scheme, which presents poor network performance, especially in high-density networks. Additionally, it is also known that when two or more concurrent LoRa transmissions occur, the one with the highest signal quality is the one to be decoded by the GW, which makes the network unfair [7]. For these reasons, in this work we explore the use of single-channel LoRa gateways operating in a channel hopping scheme. We propose a cycle-based medium access strategy for large-scale LoRa networks with multiple single-channel GWs. To increase the network fairness, each GW switches between the different channels, i.e., different combinations of transmission parameters, guaranteeing different ranges and rates of transmission so that every end-device can have its best transmission opportunity. These different channels are assigned to the end devices (ED) according to their quality of signal towards the GWs. A study on the channel allocation time was also performed to analyze its impact in the network fairness. Extensive simulation results considering different network densities, different numbers of LoRa GWs, and different channel allocation times showed that our proposal clearly outperforms the channel access scheme used by the LoRaWAN standard with respect to network goodput, packet delivery ratio, number of collisions, and access fairness.

The remainder of this paper is organized as follows. Section 2 addresses the related work regarding network management and medium access control in LoRa networks. Section 3 presents the channel hopping protocol for single-channel LoRa networks, along with the pure-ALOHA behavior for comparison purposes. Section 4 describes the simulation environment and presents preliminary results about the performance of the proposed protocol, and in Section 5 we relate an extensive study on the impact of the channel allocation time in the network performance, using goodput and fairness as performance metrics. Finally, Section 6 enumerates the main conclusions and points out directions for future work.

## 2. Related Work

Some experiments have already been executed regarding the performance of single-channel LoRa GWs in real-life environments, such as the study by Hanaffi et al. [8], in which a network with a low-cost single-channel LoRaWAN GW was tested. They analyzed the packet loss and the received signal strength of packets transmitted by sensor devices connected to the referred GW from different floors of a building. Despite the performance analysis, no strategy was presented to select the LoRa channel in use by the LoRa GW.

With the goal of improving LoRaWAN network performance, Cuomo et al. [9] proposed two different algorithms to outperform the ADR strategy. First, they proposed EXPLoRa-SF, which uses, in addition to the distance and received signal strength indicator (RSSI) values, the density of the network, i.e., the number of EDs that are in it, to assign the SFs. Next, they proposed and tested EXPLoRa-AT, a more complex algorithm than the previous one, whose goal is to assign the SFs providing a balanced distribution of the channel load among the network EDs through the equalization of the ToA. According to the tests performed, EXPLoRa-AT outperformed both ADR and EXPLoRa-SF, not only in goodput but also in data extraction rate (DER).

The work in [10] explores the idea of EXPLoRa-AT, but extends it to a multiple GW scenario. This scenario raises a situation that must be taken into account in comparison to the single GW scenario: a data packet can be received simultaneously by more than one GW. To prevent a channel being overused, giving rise to a large number of collisions, this work proposes adaptive mitigation of the air-time pressure in LoRa (AD MAIORA). This method allocates the SFs, distributing the network load by the channels and the GWs. Performance tests have shown that AD MAIORA presents a substantial improvement over the basic ADR approach.

Additionally, considering a multi-GW LoRa network, Liao et al. [11] proposed a dynamic method that selects the serving GW and allocates the most appropriate channel, considering the packet error rate (PER) for each network node. The PER value is calculated for each node using ACK/NAK signaling after every up-link transmission. Thus, the proposed method re-selects the serving GW of a node when there is a GW with better transmission conditions, and re-allocates a new SF value to the ED when the number of recent NAK signals surpasses a certain threshold. The method is based on the RSSI value between each GW and node pair to proceed to the GW re-selection, and to do the SF re-allocation, to minimize the probability of data packet collisions, the nodes are distributed in a balanced way by the several SF values. Performance tests regarding the BER and PER showed improvements of 35% and 29%, respectively, compared to the traditional SF allocation, based only on the RSSI values.

Kim et al. [12] proposed a scheme in which the LoRa gateway changes its operation channel to avoid congestion. Thus, nodes sending data marked as urgent follow the gateway as it changes its channel. However, the other nodes sending non-critical information do not change; instead, they wait until the gateway returns to the original channel to transmit again. According to the performed tests, the proposed scheme avoids congestion and improves transmission efficiency by reducing the accumulated transmission delays caused by channel congestion.

Iglesias-Rivera et al. [13] proposed a time-slotted spreading factor hopping (TSSFH) mechanism to tackle blind spots and performance issues in traditional LoRaWAN solutions. In this mechanism, nodes in a blind spot need to find a relay node connected to the gateway, resulting in a two-hop LoRa-based network that uses all available SFs, taking advantage of the orthogonality of LoRa transmissions. The communication opportunity for blind nodes increases with the listening windows opened by the relay nodes. Some performance tests of the proposed mechanism were conducted using the OMNeT++ simulator, concluding that nodes in a blind spot might reach moderate packet delivery rate values when using TSSFH in very high-density LoRa-based WANs.

TSCH-over-LoRa et al. [14] is a layer that connects the implementation of time-slotted channel hopping in Contiki-NG to a LoRa radio driver. Originally, TSCH is a synchronous MAC protocol in which all node transmissions are scheduled. The scheduling defines the timeslots each node can operate in and the channel to enable orthogonal communications. Furthermore, using a pseudo-random channel hopping sequence, TSCH is resilient to external interference and multi-path fading.

Kim et al. [15] proposed a contention-aware ADR to obtain optimal throughput. Thus, it optimizes the pure ALOHA with the gradient projection method for an ideal distribution of SFs in the network, using LoRaWAN with FHSS (frequency-hopping spread spectrum). Hence, it adjusts the SFs to increase the number of nodes using smaller values, which guarantees higher data rates.

Adelantado et al. [16] used the pseudo-random channel hopping method in LoRaWAN to tackle reliability constraints. This method allocates the transmissions for the different available channels, decreasing the number of collisions in the network. Additionally, to minimize the collision probability even further, new adaptive hopping methods were introduced.

Benkhla et al. [17] developed a mechanism called Enhanced-ADR to enhance the ADR mechanism by taking into account the position and trajectory of the EDs to reconfigure the several communication channels. The allocation model is driven by the network server, which calculates the corresponding RSSI and searches for the best RSSI interval in which it can be located to determine the most suitable channel. According to the performed tests, Enhanced-ADR improves the quality of service of the overall networks, solving the issues of ADR, such as low adaptation speed, low performance, and power consumption.

The work presented here is differentiated from the previous work for several reasons. First, instead of considering multi-channel LoRa GWs, it admits that a LoRa GW is only capable of decoding one LoRa channel at a time. This assumption is important, since it allows the possibility of a simple LoRa transceiver being used as LoRa GW. Then, the LoRa protocol here presented does not follow the channel access behavior of the LoRaWAN standard (the pure-ALOHA scheme), which is known to perform poorly in high-density scenarios. Instead, and following previous works that have shown that control packets can still be used in LoRa networks without harming the energy consumption of the EDs [18] (lower energy spent per packet delivered), we present a fair and efficient channel hopping scheme for multiple LoRa GW networks. GWs switch between different communication channels, as they can only operate one at a time, to allow all EDs in their range to successfully transmit data packets, regardless of the signal strength. Finally, the performance results achieved through simulation consider a more realistic LoRa packet capture effect [7], instead of the outdated 6 dB collision model that is still used in the literature.

## 3. A Fair MAC Channel Hopping Scheme for LoRa Networks

This work presents a MAC protocol for LoRa networks based on channel hopping, which was tested through simulation. It uses a reservation strategy, where EDs use a control packet for advertising their neighboring EDs about their intent to transmit a data packet. Moreover, each ED uses the most favorable channel available to send their data packets.

Therefore, to address every communication situation and keep a low level of complexity, +three channels were chosen, i.e., three combinations of transmission parameters, each one with a different transmission range and rate. They are:

- **Fast-Rate Channel**: Due to its short communication range capability, this channel is for EDs closer to a GW, and has excellent signal quality. It is the fastest of the three, so the EDs that use it take less time to transmit a packet. Consequently, the duty-cycle restriction time is shorter, increasing the number of transmissions allowed per ED;
- **Slow-Rate or Standard Channel**: This channel is for EDs within reach of a GW but has poor signal quality. The ToA of the data packets is the longest. The GWs use it to send synchronization packets, which will be introduced later in this chapter, as it is the one with the longest range;
- **Mid-Rate Channel**: Used by EDs with intermediate signal strength with a GW, i.e., worse than those using fast-rate but better than those using slow-rate. The ToA of the data packets is longer than in the fast-rate channel due to the more extended range, but shorter than in the slow-rate channel.

An ED only transmits data packets in its ideal channel. This is the one that guarantees the highest data rate and lowest ToA, based on the RSSI to the GWs. Before each transmission, it transmits a ready-to-send (RTS) control packet to advertise to neighboring EDs its intent. An RTS is also transmitted using the ideal channel; therefore, only the EDs in its reach that are using the same channel can decode it.

**Figure 1:**
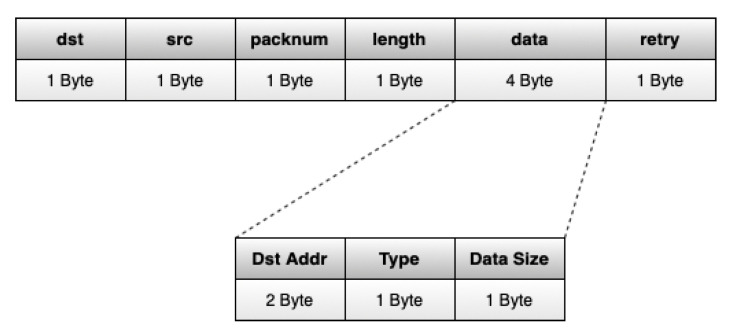
RTS packet structure.

The RTS packet structure, depicted in Figure 1, is based on the one defined by the SX1272 LoRa module support library and used in [18]. In this structure, 5 bytes are the minimum packet header, and the remaining 4 bytes are for data payload, used directly by the EDs. Of these, 2 bytes are reserved for the address of the GWs that might receive the advertised packet, 1 byte for the type of packet transmitted (control, in this case), and 1 byte for the size of the advertised packet.

The EDs can discard RTS packets based on the information regarding the targeted GWs. If none of the targeted GWs reach the ED or do not provide it with the ideal transmission rate available, the RTS is discarded. Otherwise, the ED calculates the backoff time to take, being prevented from transmitting during it. The backoff time is calculated using the data packet size described by the RTS, plus an additional time, given by a random amount of backoff slots, to reduce collisions upon backoff periods. The duration of each backoff slot is equal to the ToA of an RTS packet. After each transmission, an ED must calculate the mandatory self-restriction period, as LoRa transmissions are restricted to 1% of duty cycle. This period also includes a random number of backoff slots to desynchronize EDs that might have transmitted simultaneously the same amount of data.

As we are dealing with networks with multiple single-channel GWs, an ED can be located in an overlap zone of the GWs’ coverage. In those areas, an ED can have different ideal channels for each GW. However, it will use exclusively one of those channels, namely, the fastest one.

The GWs change their communication parameters over time to give equal chances to all EDs in their reach to transmit. When the operation channel hops from the Standard to a non-Standard, a GW sends a synchronization packet, the change mode (CM) packet, which advertises to the EDs in its reach about the non-Standard channel to which it will change and for how long it will use it. This packet is always transmitted using the Standard channel, as it has the most significant reach. The execution flow of each GW using our channel hopping protocol is shown in Algorithm 1.
**Algorithm 1:** LoRa channel hopping protocol: GW execution flow.
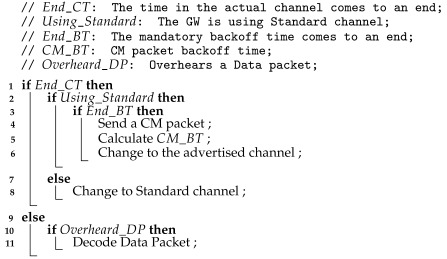


The structure of CM packets is similar to the one used in RTS packets, but with a payload of 6 bytes. Of these, 2 bytes are for the address of the transmitting GW, 1 byte for the packet type identification, 1 byte for identifying the non-Standard channel to which the GW will hop to, and 2 bytes for the period that the GW will remain in that channel.

**Figure 2:**
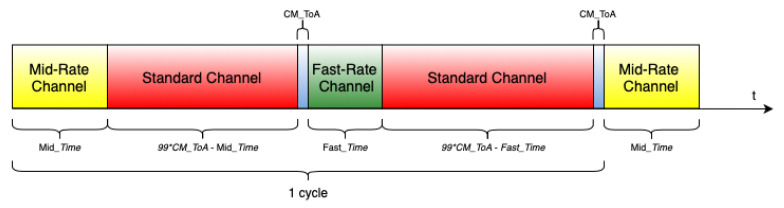
LoRa channel hopping protocol: GW hopping cycle.

As in the LoRa networks all the devices have to respect the duty-cycle restriction, the GWs have to change their operation channels accordingly. Hence, they have to wait for ninety-nine times the ToA of a CM packet between transitions to non-Standard channels. During that period, the GWs hop back to the Standard channel without further advertisement, upon the end of the non-Standard time advertised by a CM packet. As soon as the duty-cycle restriction ceases, a GW can change again to a non-Standard channel. GWs do not change to the same non-Standard channel consecutively; they alternate between Mid-Rate and Fast-Rate. Therefore, if a GW hops from the Standard to Mid-Rate channel in a first instance, in the next hop to a non-Standard channel it will chose the Fast-Rate channel, and vice versa. The GW channel hopping cycle is shown in Figure 2.

Algorithm 2 shows the complete behavior of the EDs with our channel hopping protocol. As we can see, control packets (RTS and CM) are filtered by EDs according to the targeted GWs and the used channel. Thus, EDs only consider RTS packets that advertise a transmission in their ideal channel and to the same GW, or GWs, that they use. With this, as soon as a GW in their reach changes to their ideal channel, they can transmit right away, without having to wait for the conclusion of transmissions with which they do not interfere.
**Algorithm 2:** LoRa channel hopping protocol: ED idle state execution flow.
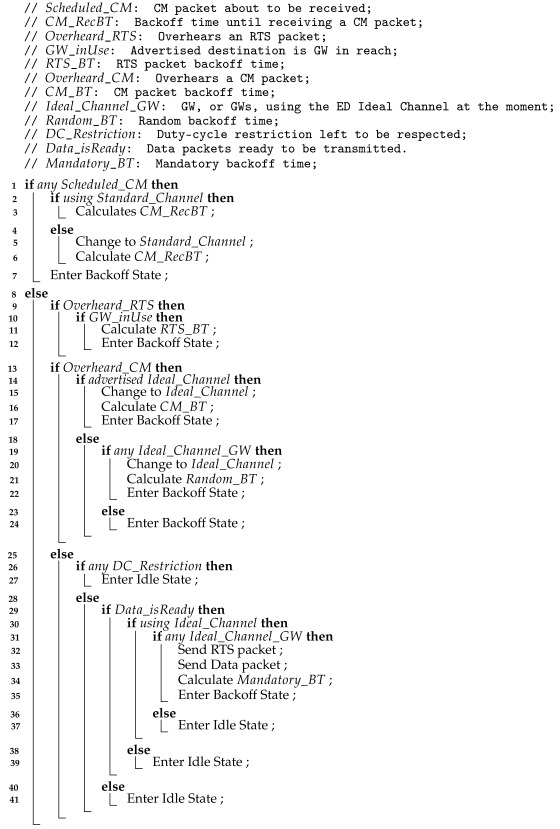


## 4. Performance Evaluation

### 4.1. Setup and Methodology

The results presented here were obtained using MATLAB simulators developed specifically to represent the behavior of each LoRa protocol: the pure-ALOHA, in which all devices use the same transmission parameters; and the channel hopping, where the EDs transmit using their most appropriate transmission channels.

The simulation time was chosen to guarantee that every ED has the opportunity to transmit, regardless of the network size. Therefore, the chosen value was 3e8 ms, approximately 83.33 h. Data packets are generated periodically so that every ED has always a packet ready to be transmitted. Regarding packet sizes, data packets have always 100 bytes, RTS packets 9 bytes, and CM packets 11 bytes, whose ToA are detailed in Table 1 (given the channel details in Table 2). The non-destructive property of LoRa was modeled following the work in [7].

For the pure-ALOHA protocol, as every device uses the same channel, the radio parameters never change, corresponding to the Standard channel ones in Table 2, as they are the ones that enable longer-range communications. The radio parameters used were based on the SX1272 LoRa module operation modes [19], and the selected frequency of operation was 868 MHz.

For each performance test, the number of EDs varied between 100 and 2000. The circumferences centered in each one of the GWs represent the maximum communication reach of each available channel. Moreover, the times allocated for the Fast-Rate and Mid-rate channels, per cycle, were 7.5 and 10 s, respectively.

**Figure 3:**
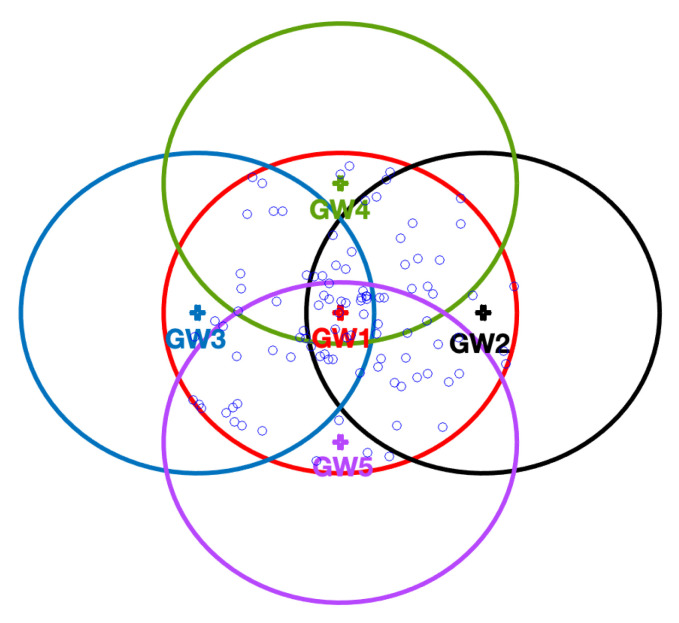
LoRa network with 5 gateways, GW1, GW2, GW3, GW4, and GW5, represented by red, black, blue, green, and purple crosses, respectively, and 100 EDs, represented as blue circles.

Figure 3 shows the positioning of the GWs in the different tested scenarios. GW1, represented as a red cross, is the only one considered in all tested scenarios. Its maximum reached using the Standard channel is represented as a red circumference; all the EDs were located in the circle limited by this circumference. Regarding the remaining GWs, 2 through 5, they were considered only for multiple GW scenarios.

Regarding the quality of each ED transmission, we decided to resort to measurements obtained by Oliveira et al. [20]. Thus, the maximum communication and signal strength ranges, for each channel, are presented in Table 3.

### 4.2. Performance Results

**Figure 4:**
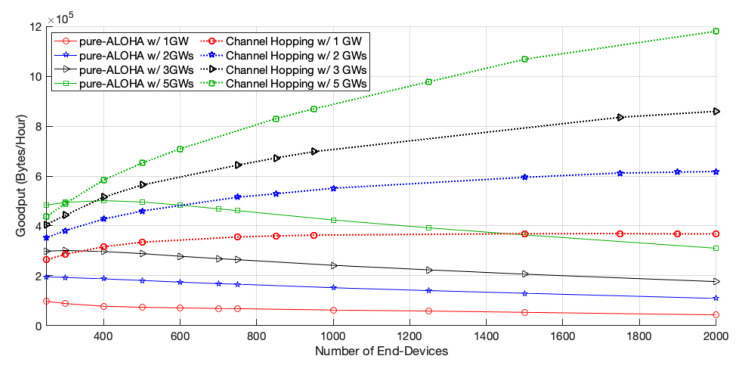
Network goodput.

Figure 4 shows the network goodput for both access protocols, for different network sizes and scenarios. With the addition of more GWs, the goodput of pure-ALOHA improves, as a higher number of EDs have a better chance to transmit their packets successfully, namely, those which are furthest from GW1. However, our scheme, when compared with the pure-ALOHA access scheme in a network of 2000 EDs, further improved the goodput by 157.07% with the installation of a second gateway (GW2), 62.01% upon including a third one (GW3), and 76.33% when five GWs were considered.

**Figure 5:**
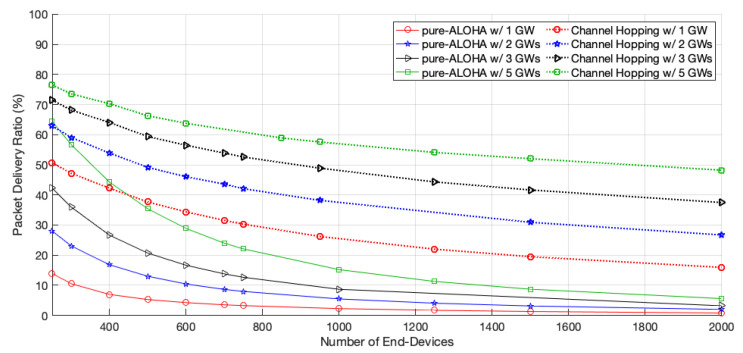
Network packet delivery ratio (PDR).

The packet delivery ratio (PDR), illustrated in Figure 5, also increased with the number of GWs; this increase was more significant for smaller networks. For the same network configuration, our scheme always outperformed the pure-ALOHA one.

**Figure 6:**
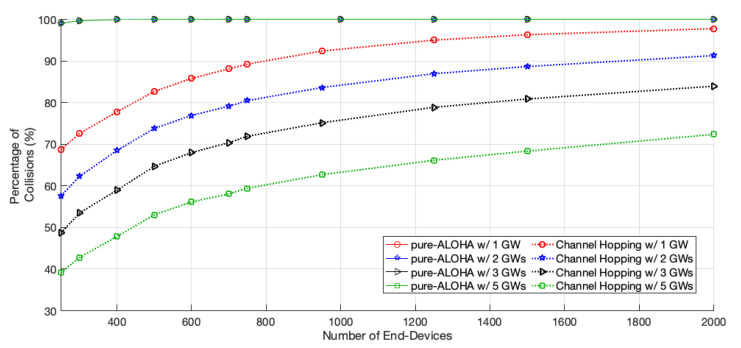
Percentage of collisions.

Regarding the collision percentage, illustrated in Figure 6, we can see that for pure-ALOHA this value is close to 100% no matter the number of gateways in the network; this is justified by the high number of EDs in the experiments, leading to a network saturation scenario. With our scheme, the percentage of collision increases with the network size. For 2000 EDs, it ranges from 72% with five GWs up to 97% with only one GW. Nevertheless, in spite of the high percentage values observed in both schemes, the network goodput is not equal to zero, thanks to the non-destructive property of LoRa.

**Figure 7:**
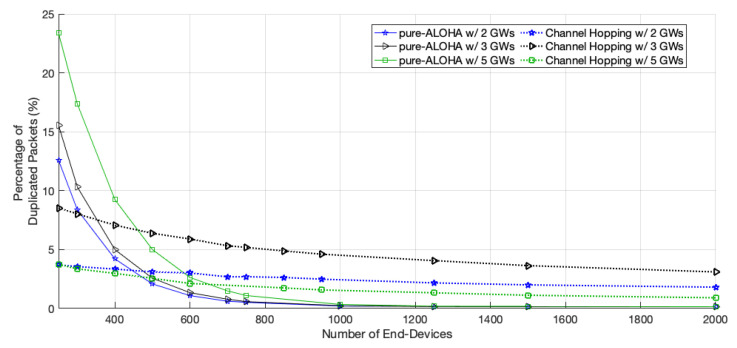
Percentage of duplicated packets among all packets received.

The percentage of duplicate packets, i.e., packets received simultaneously by more than a single GW, increases with the number of GWs, for networks with less than approximately 1000 EDs, as seen in Figure 7. From this density up, the percentages are very similar for the networks with different sizes.

**Figure 8:**
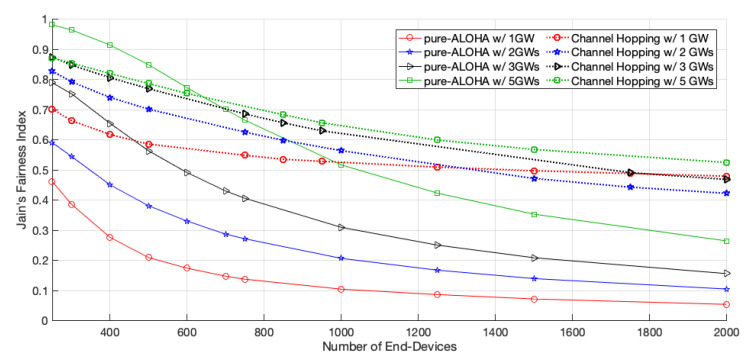
Jain’s network fairness.

As shown in Figure 8, the Jain’s network fairness of pure-ALOHA improves with the introduction of new GWs, as there is a better chance for the EDs, namely, those further from GW1, to have a better signal strength to a given GW. As the network increases, the number of collisions also increases, and the EDs with better signal quality have higher chances to deliver their packets. Therefore, the fairness decreases, regardless of the introduction of GWs to the network.

As for the channel hopping protocol, the network goodput also increases with the number of GWs. This time, the increase is more pronounced than with pure-ALOHA, as the EDs can use faster channels to transmit, and thus have more opportunities to access the medium. Moreover, as the competition to access the medium of each GW is lower, there is a better chance for the transmissions to be successful, which leads to an increase in the PDR, as shown in Figure 5. For a network size of 2000 EDs, the goodput of channel hopping increases by 68.12%, 39.14%, or 37.11%, with two, three, and five GWs, respectively, when adding GWs to the network.

**Figure 9:**
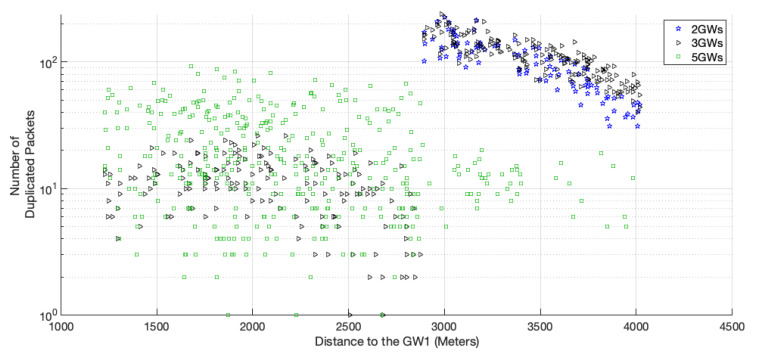
Number of duplicated packets per ED for a network with 2000 EDs using channel hopping protocol.

The percentage of duplicated packets per medium access is not as high as that obtained in pure-ALOHA for small networks; however, as the network scales up, the percentage becomes higher than pure-ALOHA’s because each transmission is more likely to be delivered, as shown in Figure 5. Moreover, with this protocol, the percentage registered for 5 GWs is the lowest of the three scenarios. The network EDs further from GW1 do not transmit duplicated packets so often, as they have closer GWs and can transmit their packets using a faster channel. This behavior is shown in Figure 9.

Regarding the network fairness of the channel hopping protocol, shown in Figure 8, for small networks, the scenario with one GW started to be the unfairer. However, for larger-scale networks, the same one became fairer and surpassed those with two and three GWs from 1313 EDs and 1800 EDs, respectively. This was due to the better transmission conditions that are guaranteed to some EDs with the addition of GWs, to the detriment of others, causing the performance differences to increase in the network. In the scenario with fiver GWs, as the GWs are distributed more equally around GW1, the network is fairer than in the others where the network is unbalanced. The EDs which use the channels with the fastest data rates can transmit packets more often than the ones that use the slowest channel. Thus, the time allocation for the channels, by the GWs, must be according to the transmission rate and density of EDs using each one of them, giving similar chances to each ED to transmit a data packet, regardless of the channel they use.

## 5. Channel Time Analysis

Up to this point, the performance analysis of both pure-ALOHA and channel hopping protocols has been focused on the number of GWs on the network. In this section, we will focus our attention on the impact of the channel time allocation on the network performance. In the previous section, the time allocated by the GWs, per cycle, to each channel was static. Thus, despite the varying number of EDs and the number of GWs covering the network, the amount of time set to each channel remained unchanged, distributing the time unfairly.

### 5.1. Channel Time Allocation for a Single GW Network

To obtain the time allocation that grants the best network fairness, we first considered a single GW network with a variable number of EDs. For each network size, several channel time distributions were evaluated in order to find the ones that grant similar chances of channel access opportunities. Table 4 presents the channel time allocations for each network size which yield the best fairness indicator. As expected, the channel time allocated to the Fast-Rate must be lower than for the other channels, regardless the network size, as the data rate is faster, and thus the EDs have more chances to access the medium.

**Figure 10:**
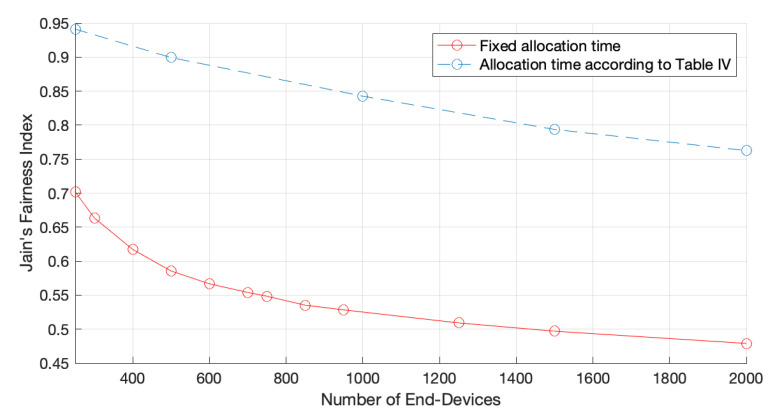
Network fairness for one GW and fair channel allocation times.

**Figure 11:**
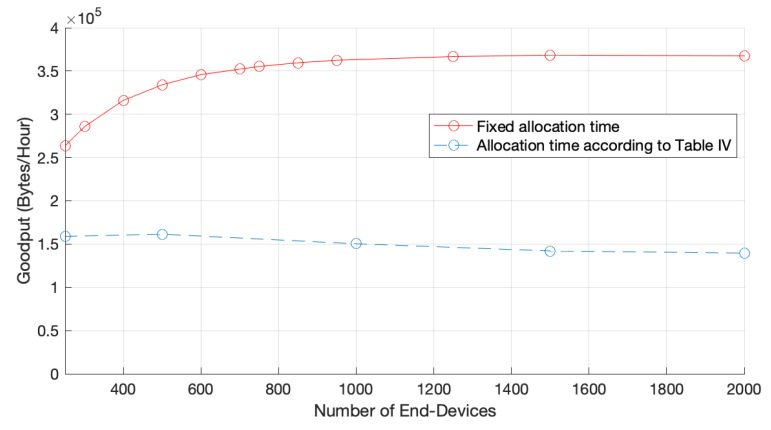
Network goodput for 1 GW and fair channel allocation times.

Figure 10 illustrates the Jain’s network fairness according to the channel allocation times presented in Table 4, where we can see that by using a customized time channel allocation time, according to the number of EDs in the network, the protocol is considerably fair when compared to a blindsided version of it, with a fixed allocation time. In particular, for a network of 2000 EDs, the network fairness index increased from 0.48 to 0.76. As explained before, by using a fair channel allocation time we are reducing the channel time allocated to the fastest channel because the data rate is higher, thereby granting the same channel access opportunities to EDs in other zones. Naturally, such a reduction has an impact on the network goodput, as illustrated in Figure 11, because EDs in faster zones are the ones that contribute more to the network goodput.

### 5.2. Channel Time Variation for Multiple GW Networks

By increasing the number of GWs, the number of EDs optimally using the Standard channel decreases. Therefore, in order to keep the network fair, the time allocated to the Standard channel must be reduced. To analyze the impact of the channel time variation in the scenario of multiple GWs, we decreased the amount of time initially given to the Standard channel and distributed to the non-Standard channels following the proportion of non-Standard times obtained for a single GW (hereafter simply denoted as percentage of decrease, or simply PoD). Therefore, the Standard channel time was decreased from 2.5% to 50%.

**Figure 12:**
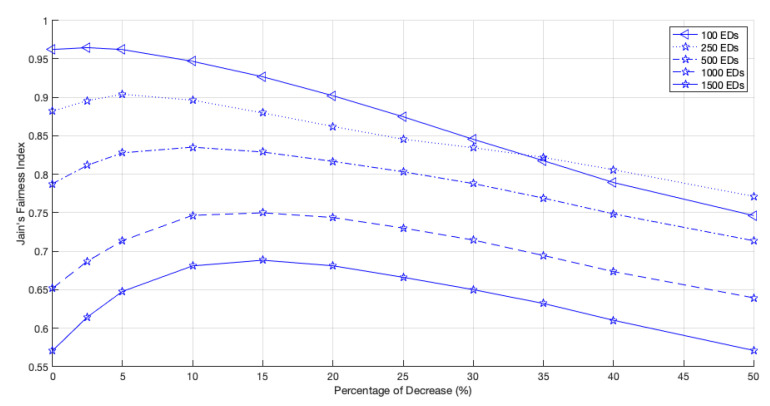
Network fairness for 2 GWs, different network sizes and different PoD.

Let us start by analyzing a network with 2 GWs. As shown in Figure 12, for large networks, the fairness index increases with the PoD until a maximum value is observed. For example, for a network size of 1500 EDs, this maximum is achieved when the PoD is around 15%, and for a network size of 100 EDs, the fairness peak is registered when the PoD is around 2.5%. When we remove more time from the Standard channel and give it to the non-Standard channels, i.e., when we increase the PoD, the fairness drops due to the lack of channel access opportunities of the EDs using the Standard channel as the optimal one.

**Figure 13:**
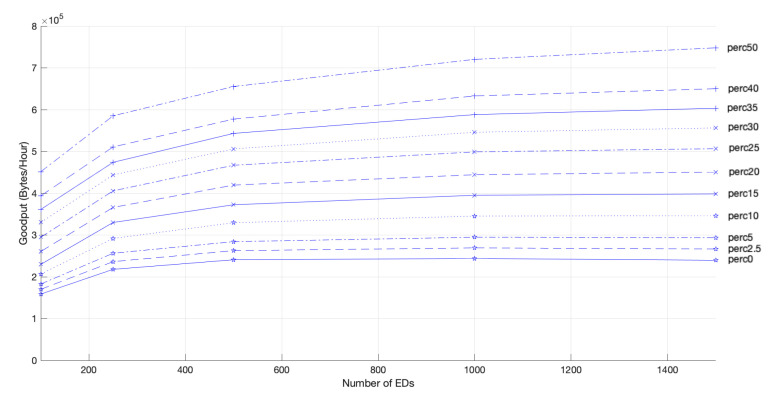
Network goodput for 2 GWs, different network sizes and different PoD.

As the EDs can transmit their packets at a higher data rate, competing for the medium more often, the network goodput improves with an increase in the PoD, as shown in Figure 13. However, as the EDs that use channels with slower data rates have fewer transmission opportunities, network fairness is negatively affected.

**Figure 14:**
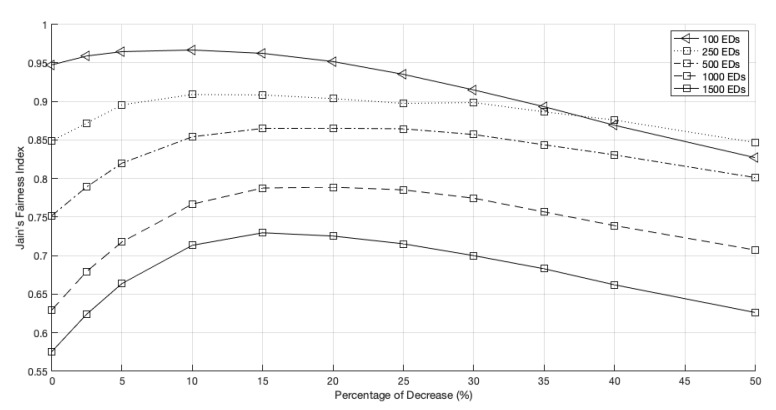
Network fairness for 3 GWs, different network sizes and different PoD.

When we install a third GW, the behavior of the network fairness, as shown in Figure 14, is identical to the prior behavior. The PoD from which the network fairness starts to decrease is about 15% when the network has 1500 EDs, the same value registered for the 2 GWs scenario. However, the peak occurrence with the network growth does not always have increasing behavior. The network with 100 EDs, regardless of initially being the fairest scenario, was surpassed when the decrease surpassed 38%. This new threshold occurred later than in the 2 GWs network (34%).

**Figure 15:**
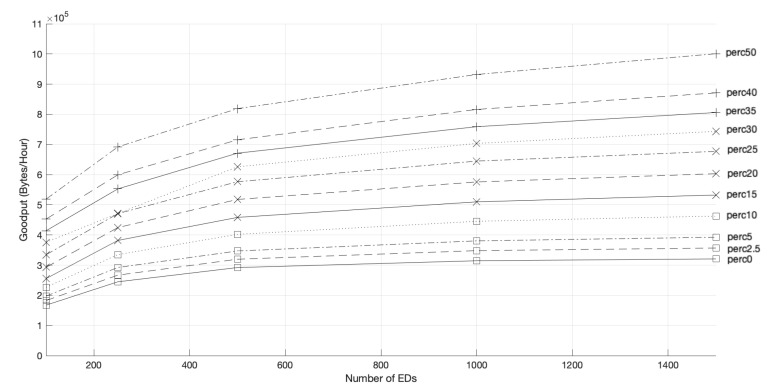
Network goodput for 3 GWs, different network sizes and different PoD.

Similarly to what happened in the scenario with 2 GWs, the network goodput for a 3 GWs scenario, illustrated in Figure 15, increased with the time allocated to the faster channels, namely, the Fast-Rate channel, which allowed the EDs to access the medium more often.

**Figure 16:**
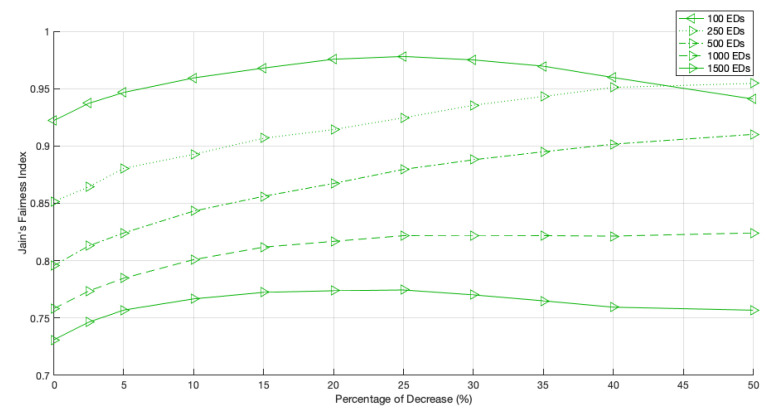
Network fairness for 5 GWs, different network sizes and different PoD.

At last, we present the results regarding the time allocation in a 5-GWs scenario. As shown in Figure 16, we can see an increase in the network fairness until the PoD of the Standard channel time surpasses 25%, for a network with 1500 EDs. This time, the fairness in less populated networks, namely, 100 EDs, increases for a PoD below 25%.

The interception between the curves of 100 EDs and 250 EDs happened for a higher PoD, approximately 44%, as shown in Figure 16. Furthermore, PoD above 40% had a more negligible influence on the fairness of networks with 1500 EDs. Like the networks with three GWs, the fairness peak does not always increase with the network growth; it starts to decrease above about 500 EDs.

**Figure 17:**
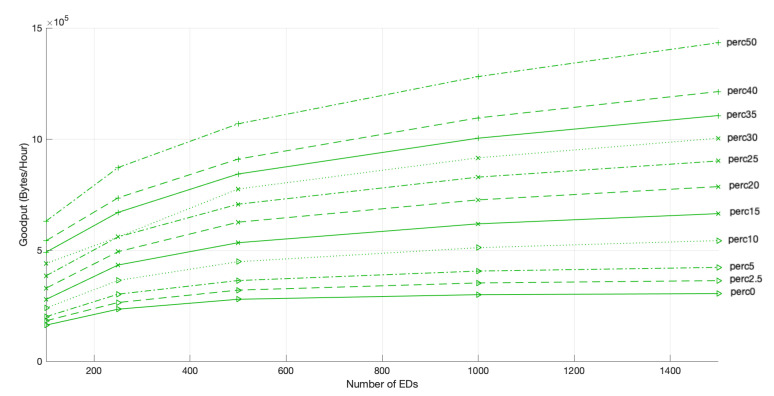
Network goodput for 5 GWs, different network sizes and different PoD.

Regarding the network goodput, the behavior is very similar to that registered for scenarios with two and three GWs: increasing with the time allocated to faster channels, as shown in Figure 17.

**Figure 18:**
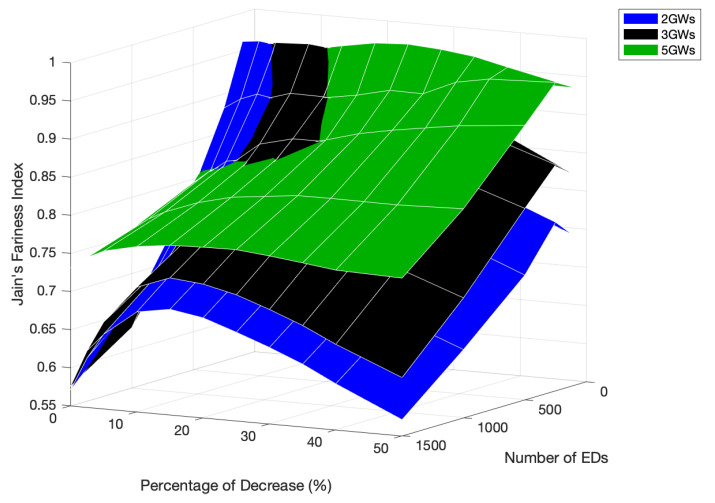
Network fairness comparison for different numbers of GWs, network sizes, and PoD.

To easily compare the network fairness, Figure 18 presents the fairness behavior for each tested scenario and the different PoD of the Standard channel time. Due to the fairer distribution of GWs by the network, the scenario with five GWs was only outperformed for percentages below 15% and in networks with under 500 EDs.

**Figure 19:**
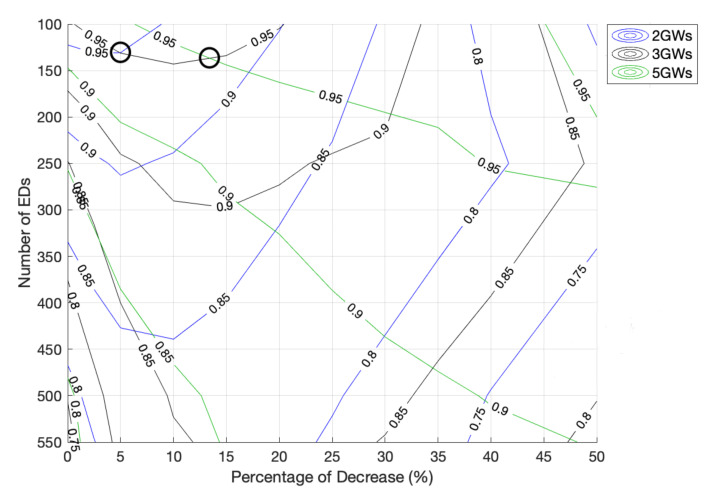
Detailed analysis of the fairness behavior regarding Figure 18.

The results also show an overlap between the three tested scenarios. First, the scenario with two GWs achieved better fairness than the others, followed by the network with three GWs. and last, the one with five GWs. This behavior is supported by Figure 19, in which the curve representing the 0.95 fairness index moves towards higher PoD as the number of GWs increases. As seen, for a network with 100 EDs, the higher fairness achieved with the original time allocation occurred with a scenario with two GWs. However, as the number of EDs increased, better fairness was guaranteed by the scenario with three GWs when the PoD was around 5%. Then, above a PoD of about 13.4%, as the 0.95 fairness lines of three and five GWs intersect, the fairness of the latter scenario became the best.

When the scenario with five GWs had 1500 EDs, the fairness peak was at 0.7745, as the PoD was equal to 25%. For the same PoD and network size, the fairness values verified for the other scenarios were 0.6662 and 0.7153, for two GWs and three GWs, respectively. This is a clear difference, which is as marked as the time allocated to faster rate channels, per GW cycle.

**Figure 20:**
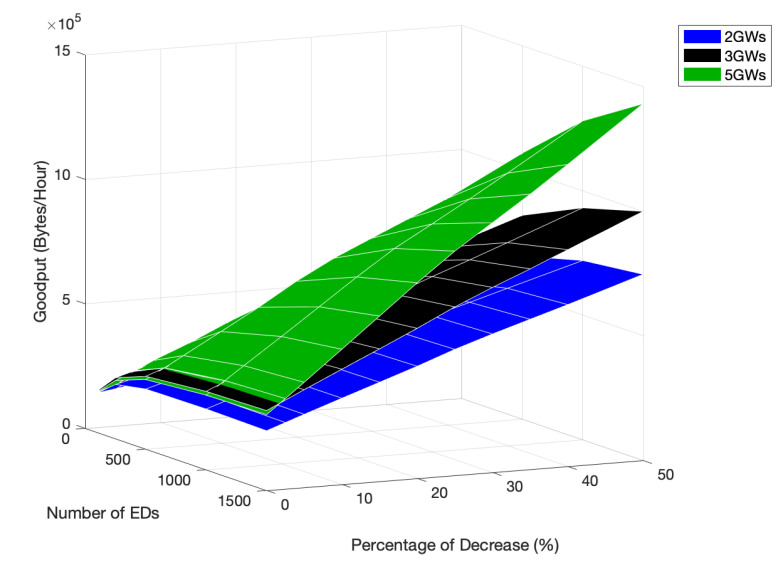
Network goodput comparison for different numbers of GWs, network sizes, and PoD.

As shown in Figure 20, by comparing the goodput for all the tested scenarios, an increase was noticeable with the number of GWs, which became more accentuated with increases in the PoD and the network size, due to the decrease in the channel access competition of each GW. Along with this, the number of EDs in the ideal channel increases in the Fast-Rate channel, which results in an increase in the number of channel accesses for the same period of time. The transmissions are faster than in slower channels, leading to smaller restriction periods.

**Figure 21:**
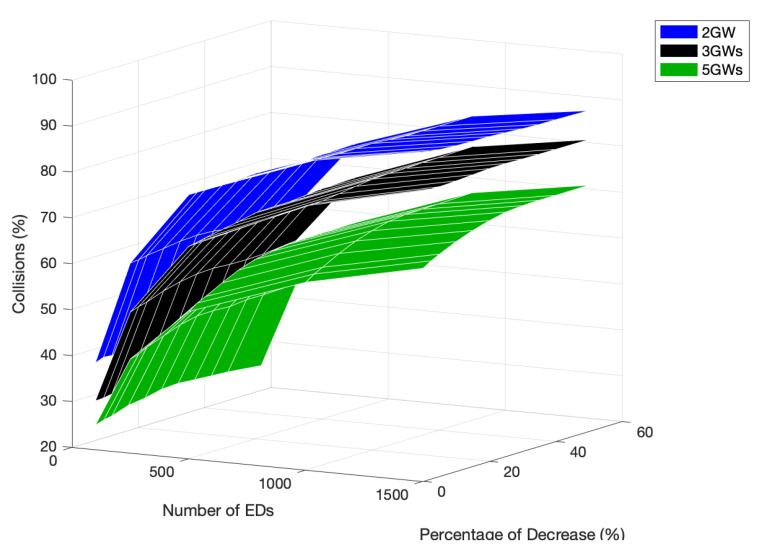
Percentage of collisions comparison for different numbers of GWs, network sizes, and PoD.

Figure 21 illustrates the percentage of collision for every tested scenario. The network with five GWs had the lowest number of channel access collisions when considering every medium access performed by the EDs. As referred to before, the main reason for this behavior is the reduction in the number of EDs sharing the channel and the lower probability of two or more EDs choosing the same time slot to transmit. Another reason is the decreasing possibility of the hidden-terminal problem. As the number of GWs increases, the EDs transmitting to a given GW, with a specific channel, are more within reach of the other EDs that also transmit to the same GW. Thus, the control packets are more likely to be received by the generality of the EDs, decreasing the number of situations where two EDs transmit at the same time because none of them receive the control packet sent by the other.

## 6. Conclusions

This study presented a new scheme for large-scale LoRa networks with multiple, low-cost single-channel GWs. The GWs switch between different operation parameters, so-called channels, to serve the EDs in their communication range, regardless of their signal strength. When comparing the developed scheme with pure-ALOHA, where every ED transmits using the same channel, the LoRa network improved regarding goodput and fairness in all tested scenarios for an arbitrary channel time allocation. The fairness for a scenario with five GWs and 2000 EDs improved from 0.2644 to 0.5245. The primary goal was to use low-cost gateways and keep the network fair, giving the same medium access opportunity to all EDs. Therefore, this paper also presented a study regarding the time allocation, per GW cycle, for the different channels, first only addressing single GW scenarios and then scenarios with multiple GWs. We concluded that the time allocation to the channel that can transmit faster must be lower to prevent an increase in the disparity of performance (goodput) between EDs using different channels. Otherwise, the network becomes unfair, giving more transmission opportunities to some EDs to the detriment of others.

In the future, to increase the performance levels of the developed protocol, we aim to add new features, such as a dynamic maximum number of backoff slots according to the number of EDs and the channels in use. We will also target the introduction of mobility in the scenario to simulate real-life scenarios more accurately and add adaptive channel periods calculated based on the number of EDs using each channel at the moment. At last, a comparison between simulated and real-life tests is planned to verify the applicability of the developed protocol.

## Figures and Tables

**Table 1:** ToA of each control packet.

	Standard Channel	Mid-Rate Channel	Fast-Rate Channel
**RTS Packet**	286.55 ms	75.52 ms	12.39 ms
**CM Packet**	307.05 ms	-	-

**Table 2:** Radio parameters characterizing the different LoRa channels used in this work.

	BW (kHz)	CR	SF	Sensitivity (dBm)
**Standard Channel**	125	4/5	10	−129
**Mid-Rate Channel**	250	4/5	9	−123
**Fast-Rate Channel**	500	4/5	7	−114

**Table 3:** Maximum communication and RSSI range for each LoRa channel.

	Range (m)	RSSI Range (dBm)
**Fast-Rate**	1210	[−100, −90]
**Mid-Rate**	2890	[−110, −101]
**Standard**	4030	[−125, −111]

**Table 4:** Channel times for a single GW scenario, per cycle.

Number of EDs	Mid-Rate Channel (ms)	Fast-Rate Channel (ms)
**100**	9205	4336
**250**	9133	2805
**500**	8774	2211
**1000**	7957	1820
**1500**	7568	1663
**2000**	7483	1628
